# Medical Items

**Published:** 1915-01

**Authors:** 


### MEDICAL ITEMS

### SPECIFY THE BRAND!

Every now and then one is forcibly reminded of the fact that the pharmaceutical market of today contains many so-called therapeutic agents of doubtful medicinal value—agents of infinite and varying potency. The point was well brought out, not so very long ago, by a certain chemist who purchased in the open market ten samples of tincture of opium in which the content of morphine varied from 2.7 to 22.8 per cent. Of three tinctures of aconite which he examined, one was found to contain 9 per cent more of aconitine than the standard required, and another 20 per cent less. Two specimens of fluid extract of the same drug contained 18.5 per cent and 25.5 per cent more, respectively, of the alkaloid than is officially required. Samples of bella-donna showed 11.5 per cent less of mydriatic alkaloids in the fluid extract of the root, and 17 per cent more in the tincture of the leaves. Some tinctures and fluid extracts of nux vomica revealed an excess of strychnine—in one case of 19 per cent.

The foregoing facts are called to mind by an announcement which is appearing in medical journals over the signature of Park, Davis & Co., bearing the title, "Fluid Extracts and Tinctures of Definite Potency," and opening with this signficant question: "When writing a prescription for a fluid extract or tincture, what assurance have you that the product dispensed will be medicinally efficient?—that it will be active, yet not too active?—that it will produce the therapeutic result that you hope for and expect?"

It is well known that Parke, Davis & Co. are authorities upon the subject of standardization, chemical and physiological, and it may be confidently asserted that the practitioner of medicine who reads and ponders what is said in the announcement referred to will find that his time has been well expended. The physician's obligation to his patient, it should be remembered, does not cease with the writing of a prescription. There remains the further duty to assure himself that trustworthy products are used in compounding that prescription. When he prescribes a fluid extract or tincture the physician owes it to his patient to specify the brand—the brand of a reliable manufacturer.

A fine lacrimal probe is the most convenient instrument for sounding the salivary ducts.—American Journal of Surgery.

A ring pessary will often give immediate relief in cases of moderate cystocele causing frequent desire to urinate.—American Journal of Surgery.

Acute infections of the kidney (pelvis) by the bacillus coli communis, however alarming the symptoms, usually subside without operative treatment.—American Journal of Surgery.

When there is bleeding from the tongue, post-operative or otherwise, and one feels reasonably sure that the hemorrhage is arterial, it can, as a rule, be easily arrested by passing the forefinger down to the epiglottis and hyoid bone and drawing the base of the tongue upward toward the chin.—American Journal of Surgery.

To secure good exposure by a transverse incision of the upper abdomen it is important that the level of the incision should not be too high. It should pass through the wide portion of the costal arch, not far from the umbilicus.—American Journal of Surgery.

In closing wounds we believe it is undesirable to pass skin sutures through the subcutaneous fat, which is easily liquefied and infected. The fat layer, however thick, is amply coated by the closure of the skin and the application of the dressing.—American Journal of Surgery.

For the closure of abdominal and other wounds fine sutures applied close to the skin edge and only through the skin thickness produce little or no scarring. Those taking large "bites" of the skin and subcutaneous fat, so commonly used, leave ugly "stepladder" or "centipede" scars. Tension sutures, too, also used by many surgeons, are rarely necessary, even after breast amputations.—American Journal of Surgery.

A very satisfactory operation for umbilical hernia in cases where the ring is not of huge size and the abdominal wall not attenuated, consists of splitting open, like an oyster, each rectus sheath at the ring and for a greater or less distance in the linea alba above and below, and suturing together the posterior and anterior layers of the two sheaths in separate layers. If the rectus muscles are not separated, as they so often are in multiparous women, the aponeurosis sutures are sufficient to approximate them. If they are separated, it is undesirable to stretch and tear them by approximating muscle sutures. The operation can be performed through a vertical or a transverse incision. The former is the more convenient and the less disfiguring.—American Journal of Surgery.

---

### CONVALESCENCE.

The secret of prompt recovery from many a serious illness will be found in the prompt institution of tonic treatment. The resulting uplift is often all that is needed to enable the body to reestablish a nutritional balance and develop adequate resistance.

Thus, after the acute diseases, such as typhoid fever, pneumonia, pleurisy, influenza or those requiring surgical operations, like appendicitis, intestinal ailments, utero-ovarian ailments and so on, the return to health often hinges on the thought and care given to restorative treatment. If a reconstructive like Grays' Glycerine Tonic Comp. is used, the result is rarely if ever in doubt. Unlike many remedies used to promote convalescence, Gray's does not whip up weakened forces. On the contrary, it aids and reinforces them by increasing the power and capacity of physiologic processes throughout the body. Thus the appetite is improved, digestive and absorptive functions are activated and the resulting improvement in cellular nutrition insures a notable gain in vitality and strength. Weakness and debility vanish as vitality and strength appear. This tells why "Gray's" is so useful and effective after the acute diseases.

---

### PITUITARY EXTRACT IN OBSTETRICAL PRACTICE.

Physicians who are employing pituitary extract in cases of delayed parturition will be interested in this excerpt from an announcement by Parke, Davis & Co., which appears in the December issue of a contemporary:

